# Supplementary material for: HIVprotI: an integrated web based platform for prediction and design of HIV proteins inhibitors
Source: J Cheminform. 2018 Mar 9;10:12. doi: 10.1186/s13321-018-0266-y (PMC5845081; doi:10.1186/s13321-018-0266-y)
Supplement: Supplementary file 2 — Additional file 2. Source code of HIVProtI web server. [file 13321_2018_266_MOESM2_ESM.zip › HIVprotI_Source-code/hivprotI/weka-3-6-6/doc/weka/filters/SimpleFilter.html]

SimpleFilter


---


|  |  |  |
| --- | --- | --- |
| **Overview** \| **Package** \| **Class** \| **Tree** \| **Deprecated** \| **Index** \| **Help** \| **Weka's home** | |  |
| **PREV CLASS**   **NEXT CLASS** | **FRAMES**    **NO FRAMES**     **All Classes** |  |
| SUMMARY: NESTED \| FIELD \| CONSTR \| METHOD | DETAIL: FIELD \| CONSTR \| METHOD |  |


---


## weka.filters Class SimpleFilter

```
java.lang.Object
  weka.filters.Filter
      weka.filters.SimpleFilter
```

**All Implemented Interfaces:**: java.io.Serializable, CapabilitiesHandler, OptionHandler, RevisionHandler

**Direct Known Subclasses:**: SimpleBatchFilter, SimpleStreamFilter

---

``` public abstract class SimpleFilter extends Filter implements OptionHandler ```

This filter contains common behavior of the SimpleBatchFilter and the
SimpleStreamFilter.

**Version:**
:   $Revision: 1.4 $

**Author:**
:   FracPete (fracpete at waikato dot ac dot nz)

**See Also:**: `SimpleBatchFilter`, `SimpleStreamFilter`, Serialized Form

---

**Constructor Summary**

`SimpleFilter()`


| **Method Summary** | |
| --- | --- |
| `java.lang.String` | `debugTipText()`             Returns the tip text for this property |
| `boolean` | `getDebug()`             Returns the current debugging mode state. |
| `java.lang.String[]` | `getOptions()`             Gets the current settings of the filter. |
| `abstract  java.lang.String` | `globalInfo()`             Returns a string describing this classifier. |
| `java.util.Enumeration` | `listOptions()`             Returns an enumeration describing the available options. |
| `void` | `setDebug(boolean value)`             Sets the debugging mode |
| `boolean` | `setInputFormat(Instances instanceInfo)`             Sets the format of the input instances. |
| `void` | `setOptions(java.lang.String[] options)`             Parses a list of options for this object. |

**Methods inherited from class weka.filters.Filter**

`batchFilterFile, batchFinished, filterFile, getCapabilities, getCapabilities, getOutputFormat, getRevision, input, isFirstBatchDone, isNewBatch, isOutputFormatDefined, main, makeCopies, makeCopy, numPendingOutput, output, outputPeek, toString, useFilter, wekaStaticWrapper`

**Methods inherited from class java.lang.Object**

`equals, getClass, hashCode, notify, notifyAll, wait, wait, wait`

**Constructor Detail**

### SimpleFilter

```
public SimpleFilter()
```


**Method Detail**

### globalInfo

```
public abstract java.lang.String globalInfo()
```

:   Returns a string describing this classifier.

    :   **Returns:**: a description of the classifier suitable for displaying in the explorer/experimenter gui

---


### listOptions

```
public java.util.Enumeration listOptions()
```

:   Returns an enumeration describing the available options.

    :   **Specified by:**: `listOptions` in interface `OptionHandler`
    :   **Returns:**: an enumeration of all the available options.

---


### setOptions

```
public void setOptions(java.lang.String[] options)
                throws java.lang.Exception
```

:   Parses a list of options for this object.
    Also resets the state of the filter (this reset doesn't affect the
    options).

    :   **Specified by:**: `setOptions` in interface `OptionHandler`
    :   **Parameters:**: `options` - the list of options as an array of strings **Throws:**: `java.lang.Exception` - if an option is not supported **See Also:**: `reset()`

---


### getOptions

```
public java.lang.String[] getOptions()
```

:   Gets the current settings of the filter.

    :   **Specified by:**: `getOptions` in interface `OptionHandler`
    :   **Returns:**: an array of strings suitable for passing to setOptions

---


### setDebug

```
public void setDebug(boolean value)
```

:   Sets the debugging mode

    :   **Parameters:**: `value` - if true, debugging information is output

---


### getDebug

```
public boolean getDebug()
```

:   Returns the current debugging mode state.

    :   **Returns:**: true if debugging mode is on

---


### debugTipText

```
public java.lang.String debugTipText()
```

:   Returns the tip text for this property

    :   **Returns:**: tip text for this property suitable for displaying in the explorer/experimenter gui

---


### setInputFormat

```
public boolean setInputFormat(Instances instanceInfo)
                       throws java.lang.Exception
```

:   Sets the format of the input instances.
    Also resets the state of the filter (this reset doesn't affect the
    options).

    :   **Overrides:**: `setInputFormat` in class `Filter`
    :   **Parameters:**: `instanceInfo` - an Instances object containing the input instance structure (any instances contained in the object are ignored - only the structure is required). **Returns:**: true if the outputFormat may be collected immediately **Throws:**: `java.lang.Exception` - if the inputFormat can't be set successfully **See Also:**: `reset()`


---


|  |  |  |
| --- | --- | --- |
| **Overview** \| **Package** \| **Class** \| **Tree** \| **Deprecated** \| **Index** \| **Help** \| **Weka's home** | |  |
| **PREV CLASS**   **NEXT CLASS** | **FRAMES**    **NO FRAMES**     **All Classes** |  |
| SUMMARY: NESTED \| FIELD \| CONSTR \| METHOD | DETAIL: FIELD \| CONSTR \| METHOD |  |


---
